# Editorial for the Special Issue on “Human Biomonitoring in Health Risk Assessment: Current Practices and Recommendations for the Future”

**DOI:** 10.3390/toxics11020168

**Published:** 2023-02-10

**Authors:** Christophe Rousselle

**Affiliations:** The French Agency for Food, Environmental and Occupational Health & Safety (ANSES), 14 Rue Pierre et Marie Curie, 94701 Maisons-Alfort, France; christophe.rousselle@anses.fr

In most health risk assessment (HRA) frameworks for chemicals, the default approach for exposure assessment is to estimate the intake from different sources and different routes of exposure. These assessments are often made separately and then added when aggregate exposure scenarios are considered. Various uncertainties are associated with this approach and, depending on the scope of the assessment; it may over- or under-estimate the internal exposure. Having access to data on the internal exposure generated by human biomonitoring gives a complete picture of human exposure and can be used to enhance a chemical risk assessment by providing information on actual human exposure via multiple exposure pathways. An understanding of the contribution that different exposure routes make to the overall exposure (taking into account differences in exposure modifiers, such as age and gender, as well as profession) delivers a starting point for deciding on risk management measures under legislative silos.

Human biomonitoring (HBM) is an important and useful tool for assessing the internal exposure of humans resulting from aggregated exposure to chemicals. HBM can also provide a better estimate of exposure close to the target organ. The inclusion of HBM data could improve HRAs for the general population and workers. Although there are still a number of obstacles that hinder the use of HBM data in HRAs, the growing availability of HBM data offers an opportunity to improve and refine RAs.

This Special Issue intends to illustrate, using case studies, how HBM data could be used to better estimate the internal exposure and resulting risks. Case studies either on exposure from the use of consumer products (cosmetic products, non-food products, etc.) or from exposures via food or water, in the general population or among workers, have contributed to better identify the hurdles that prevent a broader use of HBM data in RAs. New tools such as physiologically based pharmacokinetic (PBPK) models, derivations of health-based guidance values, new approaches for integrating HBM with in vitro/in silico data, and adverse outcome pathways (AOP), through more accurate data on actual internal exposure, could improve HRAs.

The Special Issue “Human Biomonitoring in Health Risk Assessment: Current Practices and Recommendations for the Future”” collected and published 15 contributions focusing on the generation of biomonitoring data on chemicals in different populations, including workers, and on their use in a risk assessment context. The published papers comprise 4 reviews and 11 original articles, among which 8 reported results from the HBM4EU Initiative https://www.hbm4eu.eu/ accessed on 7 January 2023).

Measuring the concentration of a chemical in urine or blood could also be used as a more robust surrogate marker of systemic exposure compared to calculations based on external exposure scenarios. In their review, Willenbockel et al. (2022) [1] quantified the systemic exposure of humans exposed to pesticides used on specific cultures such as tree-grown produce, vines or hops. The analysis revealed that exposure was mainly driven by the application of pesticides and re-entry work, resulting in a higher exposure of operators and workers than of residents and bystanders. In nearly all cases, the systemic exposure was below the relevant toxicological reference values.

Blood and urine are the most common matrices investigated in HBM studies as they are easily accessible. However, other biological matrices may be of some interest as they may constitute a better proxy for systemic exposure. In their review, Moriceau et al. (2023) [2] compared the serum/adipose tissue ratio for persistent organic pollutants (POPs) and sought to identify key factors that could explain why for some compounds the ratio differed from 1. All included studies reported high variability in the partition coefficients of POPs and the authors concluded that further research is still needed to better investigate the partitioning of POPs. 

In broncho-alveolar lavages (BALs) it is also interesting to analyze inhaled bio-persistent particles, particularly when following occupational exposure. Forest et al. (2022) [3] investigated the relationship between the biomonitoring of nanoparticles in BALs, interstitial lung diseases and occupational exposure to these unintentionally released particles. Their results strengthen the array of presumptions on the contribution of some inhaled particles (from nano- to submicron-sized) to some idiopathic lung diseases. 

To interpret the measured concentrations, reference values are necessary. Two types of references values were illustrated in this Special Issue health-based guidance values (HBM-GVs) and a background range in the general population. Meslin et al. (2022) [4] derived HBM-GVs for bisphenol S (BPS) and used results from HBM4EU studies to assess the risk due to the exposure to BPA and BPS in Europe, for workers and the general population. In the same way, Lamkarkach et al. (2022) [5] derived HBM-GVs for dimethylformamide (DMF) for exposed workers. A large database on DMF exposure from studies conducted at workplaces provided dose–response relationships between biomarker concentrations and health effects. 

Measuring effect biomarkers and studying their associations with health outcome data could also provide insight into the extent to which chemicals and substitutes have an impact on health. Malondialdehyde (MDA) is an end-product of lipid oxidation in our cells and is present in all biological matrices, including exhaled breath condensate (EBC) and urine. Following a systematic literature review and meta-analysis, Turcu et al. (2022) [6] proposed reference values for MDA in EBC. However, defining the distribution of MDA in EBC measured in reference populations still represents a challenge due to the low number of available studies, different analytical methods, and the questionable methodological quality of many studies. In urine, following a systematic literature review and meta-analysis, Toto et al. (2022) [7] sought to establish urinary MDA concentration ranges for healthy adult populations based on reported values in the available scientific literature. The proposed urinary MDA values should be considered preliminary, as they are based mostly on moderate- to low-quality studies. Bergamaschi et al. (2022) [8] performed a pilot biomonitoring study in workers from a paint production plant exposed to pigment-grade titanium dioxide (TiO_2_). They assessed pro-inflammatory cytokines (IL-1β, TNF-α, IL-10, and IL-17), surfactant protein D (SP-D) and Krebs von den Lungen-6 glycoprotein (KL-6) in EBC. Their findings suggest the need for an integrated approach relying on both personal exposure and biomarker assessment to improve the hazard characterization in occupational settings.

HBM data can also be used to perform risk assessments and suggest risk management measures if the internal exposure exceeds the HBM-GVs. Using BP-3 as an example, Rousselle et al. (2022) [9] investigated the benefits and limitations of the use of external versus internal exposure data to explore the usefulness of HBM to support the risk assessment of cosmetic ingredients. The results showed that both approaches did indicate a risk to human health under certain levels of exposure. They also highlighted the need for more robust exposure data on BP-3 and other cosmetic ingredients, and a standardized framework for incorporating HBM data in the risk assessment of cosmetic products. In the same way, Huuskonen et al. (2022) [10] demonstrated, by using the example of ortho-toludine, how HBM data can be used to assess cancer risks for workers and the general population. The urinary mass-balance methodology and generic exposure reconstruction PBPK modelling were both used to estimate the external intake levels corresponding to the observed urinary levels. Domínguez-Morueco (2022) [11] performed a HRA to estimate the risk associated with methylmercury exposure of vulnerable European populations using HBM data. As many data were missing to make a proper assessment, the authors concluded that further HRA refinement is needed with coordinated, widespread HBM data to account for the differences in European exposure and associated risks, so that interventions can be applied to protect vulnerable citizens. Plichta et al. (2022) [12] assessed the risk of dietary exposure to organophosphorus flame retardants (OPFRs) in children using HBM data and estimated how much dietary intake may contribute to the total exposure. The estimated exposure to OPFRs indicates a minimal health risk based on the current knowledge of the available exposure, kinetic and toxicity data.

When no HBM data are available, for example, in the case of prospective assessments, the internal dose of a pesticide in human can be estimated by the expected residue levels in food. Tarrazona et al. (2022) [13] compared consumers’ internal exposure to chlorpyrifos based on the urinary marker 3,5,6-trichloro-2-pyridinol (TCPy), using two sources of monitoring data: monitoring of the food chain from the EU program and biomonitoring of European citizens from the HBM4EU initiative, supported by a literature search. Both methods confirmed a drastic reduction in the exposure levels from 2016 onwards. Finally, in Tarvares et al. (2022) [14], a mixture risk assessment based on published HBM data on Cr (VI), Ni and/or PAH occupational co-exposure in Europe was performed and in some situations the sum of risk quotients (SRQ) exceeded 1, which means that risk cannot be excluded, for example, in the welding or waste incineration sectors.

HBM can also be used to assess the impact of management measures taken to reduce external exposures. In their study, Buonaurio et al. (2022) [15] evaluated the effects of traffic on human health comparing biomonitoring data measured during the COVID-19 lockdown, when restrictions led to a 40% reduction in airborne benzene in Rome and a 36% reduction in road traffic, to the same parameters measured in 2021. Their results confirmed a decrease in succinic acid, a product of the Krebs cycle promoting inflammation.

To conclude, a better understanding of population exposure and the exposure of vulnerable groups against health-based human biomonitoring guidance values, provides the basis for effective risk management aimed at reducing the impacts on health. Europe’s zero-pollution agenda should depart from an understanding of how European citizens’ bodies are polluted with synthetic chemicals and make the reduction in chemical body burden and the associated health impacts a key priority.

We would like to thank all the authors for submitting their original contributions to this Special Issue. We greatly appreciate the support of all the reviewers who spent time evaluating and improving the quality of the manuscripts. We would also like to thank the editors of *Toxics* for their kind invitation, and Selena Li of the *Toxics* Editorial Office for her precious support.

## Data Availability

Not applicable.
